# Supplementary material for: Reconstruction and analysis of the transmission network of African swine fever in People’s Republic of China, August 2018–September 2019
Source: Epidemiol Infect. 2024 Jan 29;152:e27. doi: 10.1017/S0950268824000086 (PMC10894904; doi:10.1017/S0950268824000086)
Supplement: Akhmetzhanov et al. supplementary material [file S0950268824000086sup001.docx]

**Supplementary Figures and Tables**

Supplementary Figure 1: First appearance and last outbreak by province of China. (A) shows start date of the first outbreak and end date of the last outbreak–if declared–for each province (black triangles). The red circles indicate the start dates of the other outbreaks. (B) and (C) are heatmaps of first outbreak start date (B) and last outbreak end date (C) by province. The grey areas indicate provinces with ongoing outbreaks as of 9 September 2019.


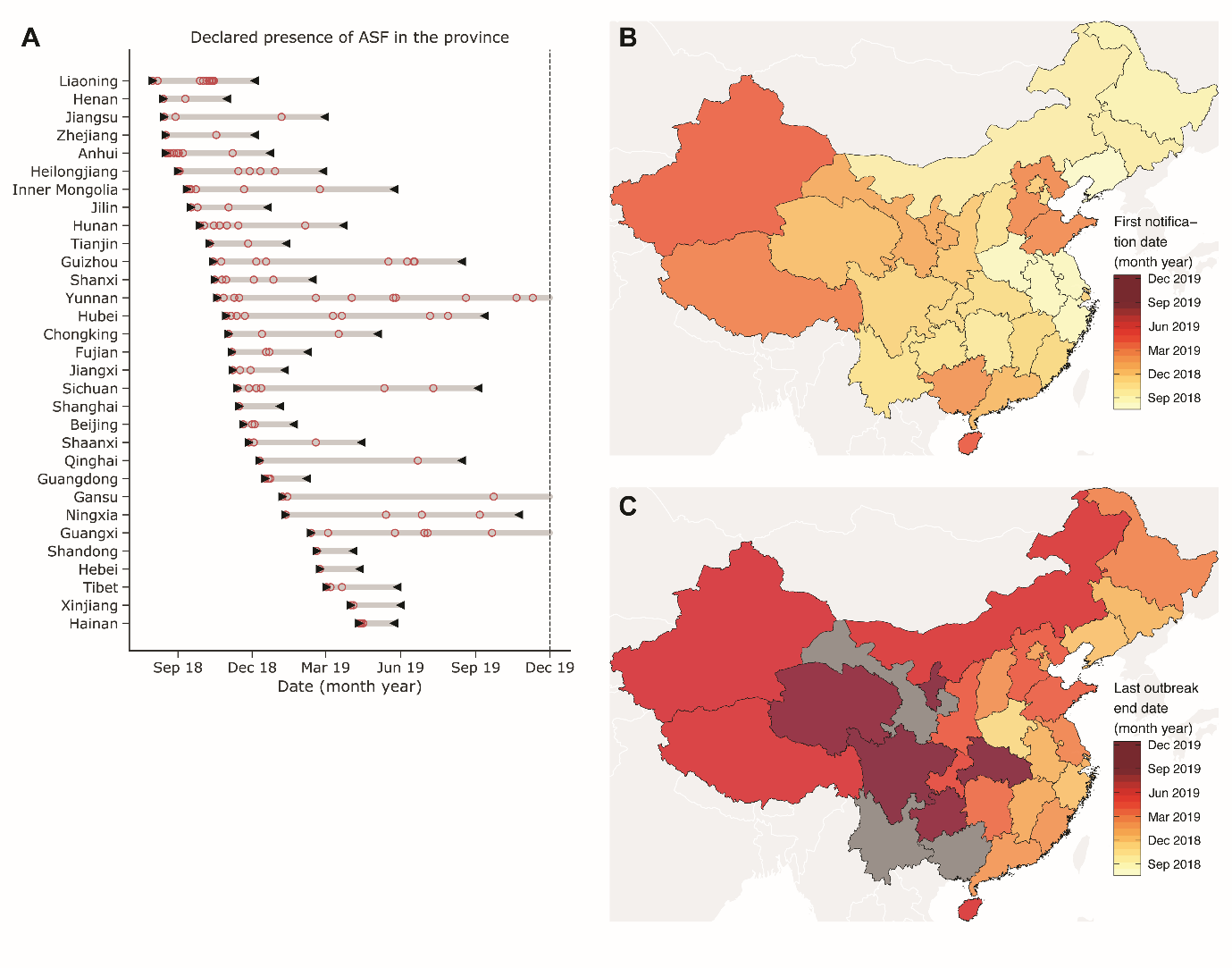


Supplementary Figure 2: Weekly number of reported and terminated outbreaks of African swine fever (ASF) factorized by type (A) or size of the unit (B). Epidemic curves by (A) type of unit and (B) size of the unit are shown. The coloured bars indicate the different types and sizes of infected units in China from July 2018–September 2019.


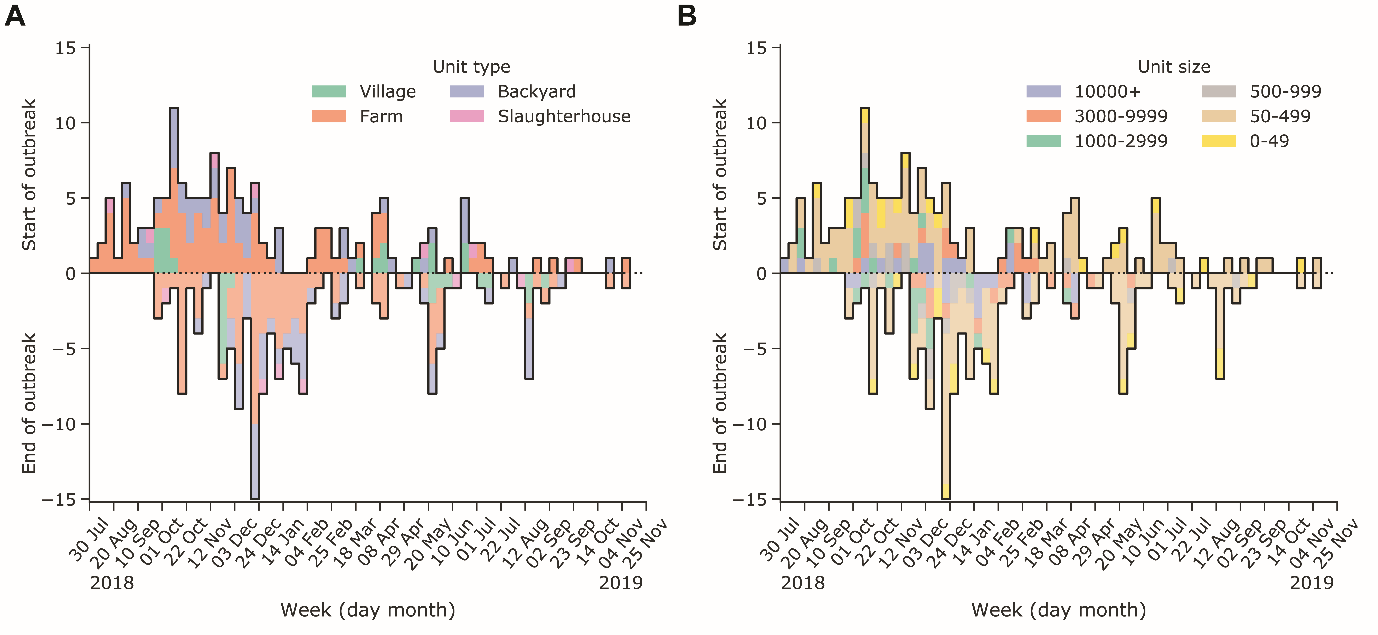


Supplementary Figure 3: Reporting delay by characteristic of African swine fever (ASF) infected unit. Distribution of the reporting delay by (A) province, (B) size of the unit and (C) type of the unit are shown. Reporting delay is calculated using outbreak start and report dates, as released by the World Organization for Animal Health (OIE). The bar colours represent the various characteristics of the ASF infected units.


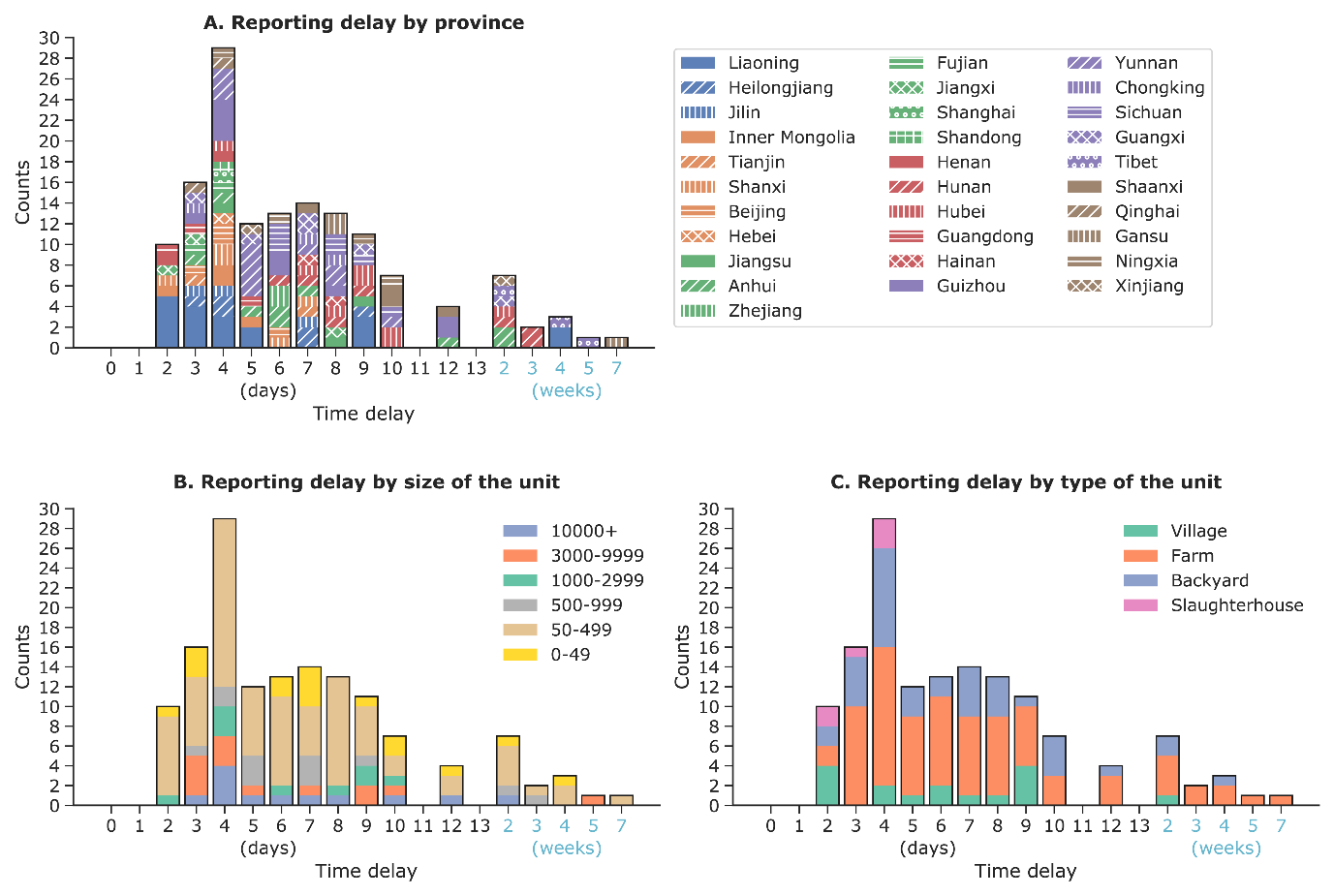


Supplementary Figure 4: Outbreak linkage by nearest neighbour or exponential function algorithms (A, B) or by equal probability algorithm (C, D). (A) and (C) show the number of outbreaks with detected links (solid bars) vs total number of outbreaks by week of outbreak start. (B) and (D) show the fraction of outbreaks with detected links over the preceding six weeks by week of outbreak start. The dashed horizontal red line indicates a 80% fraction of detected links.


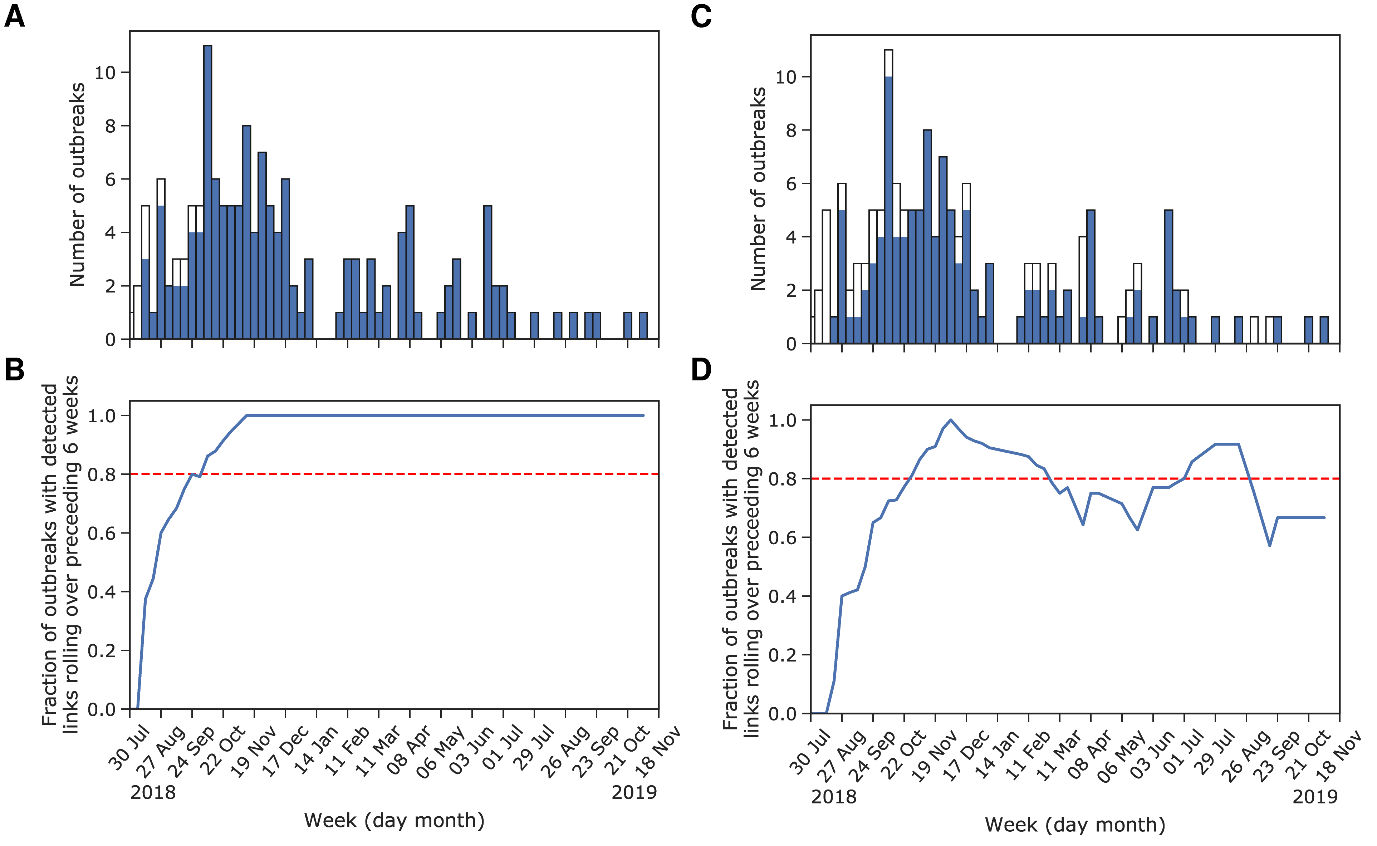


Supplementary Figure 5: Estimated weekly reproduction number via exponential function algorithm using four different effective transmission distances $\bar{\boldsymbol{d}}$. The lines and grey shading represent the reproduction number by week including 95% credible intervals. The threshold number of one is represented with a dashed line.


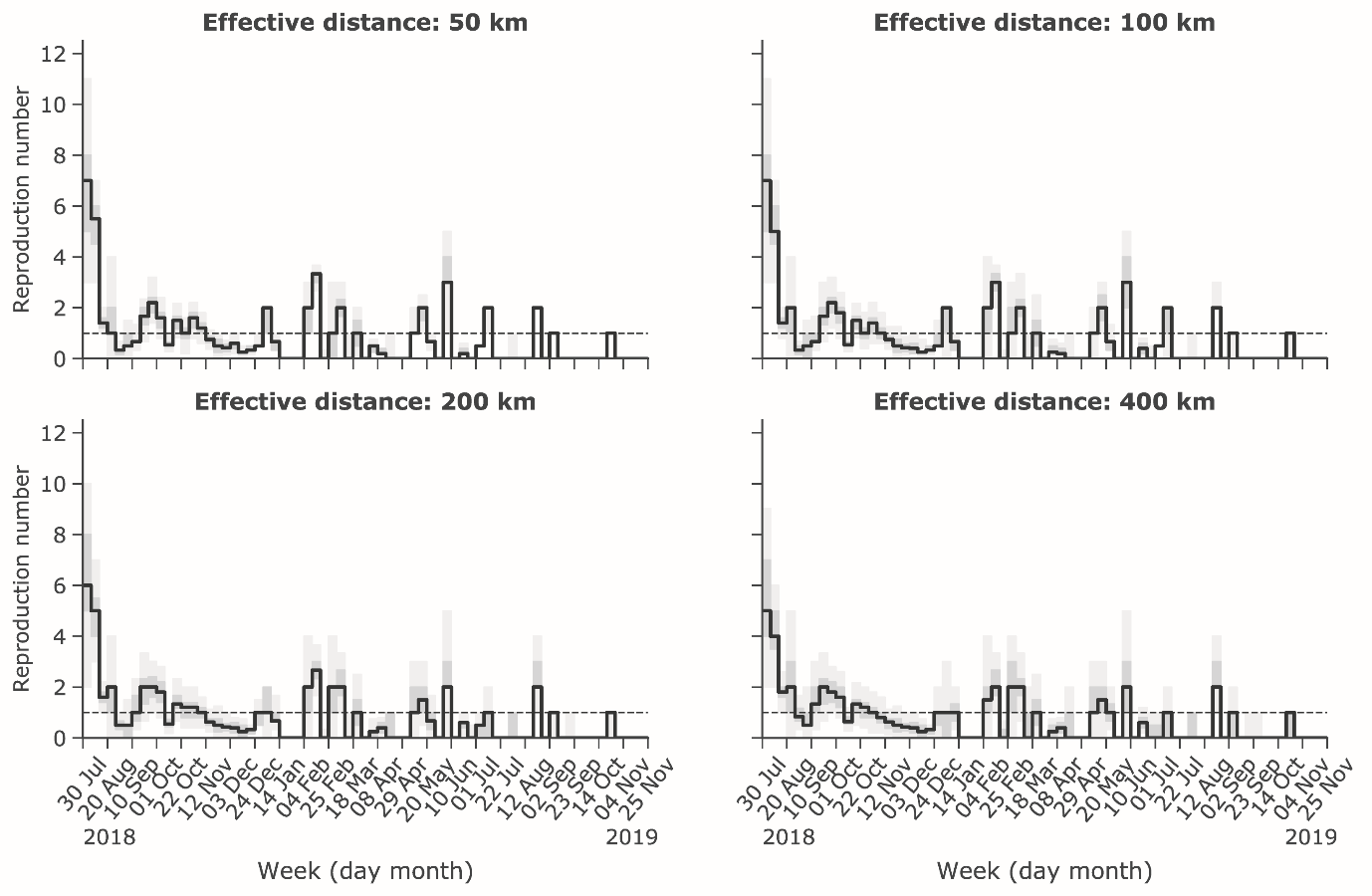


Supplementary Figure 6: Estimated weekly reproduction number via equal probability algorithm, using four different effective transmission radii $\bar{\boldsymbol{r}}$. The lines and grey shading represent the reproduction number by week including 95% credible intervals. The threshold number of one is represented with a dashed line.


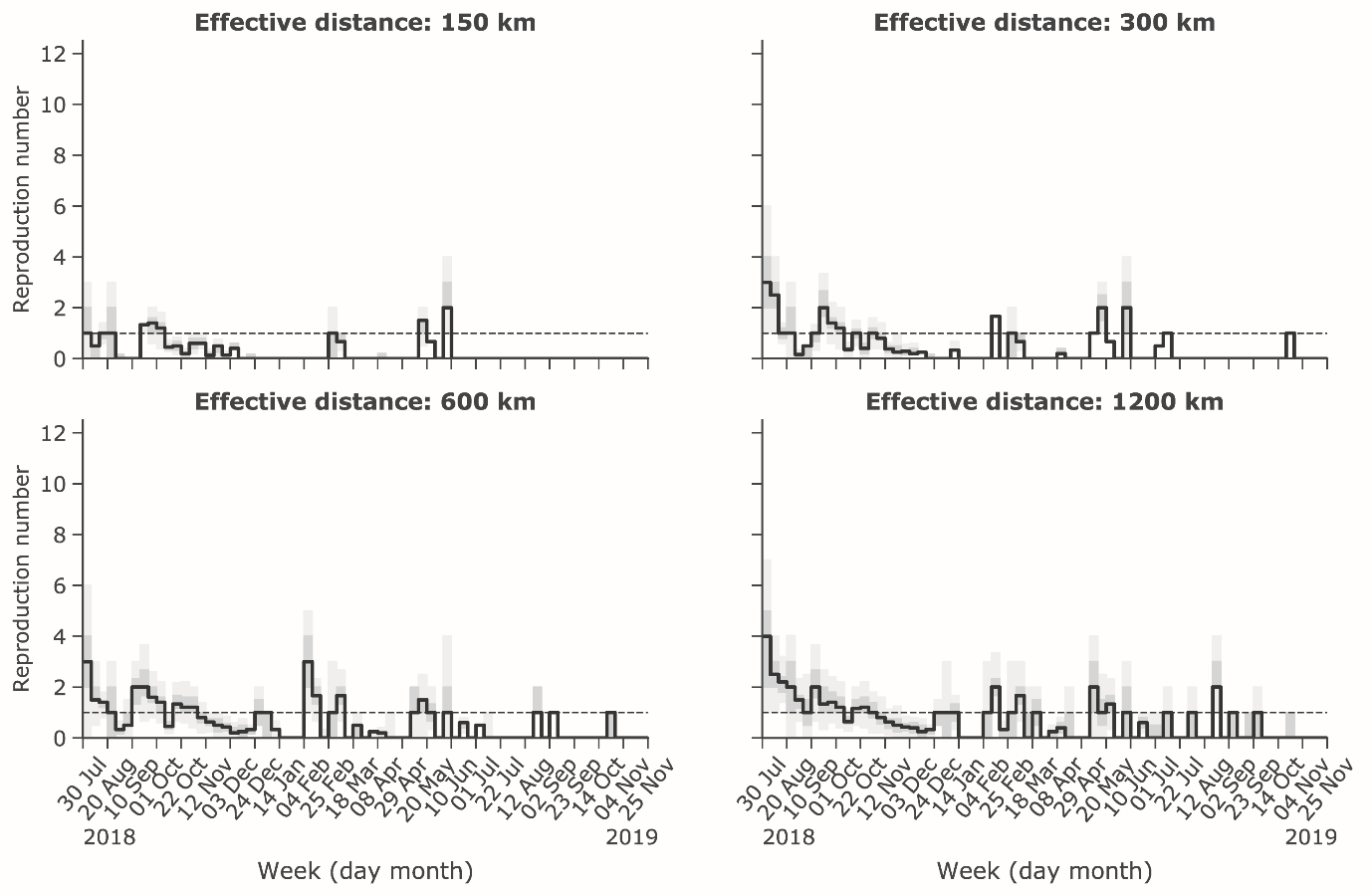


Supplementary Figure 7: Analysis of a historical epidemic of ASF in Odessa region of Ukrainian Soviet Socialistic Republic (SSR) of the former Soviet Union, 1977, to determine the generation time distribution. (A) shows the map of Ukrainian SSR with highlighted locations of the reported outbreaks. (B) depicts the transmission routes determined as most likely by investigation of the epidemic. The left panel is respectively to the number of infected units, the right panel is respectively to the number of culled pigs: market sell of port products affected only backyard private farms. (C) shows the fitted multi-generational model to the data, whereas (D) the resulting generation time distribution. Shaded area in green indicates 95% credible interval.

**
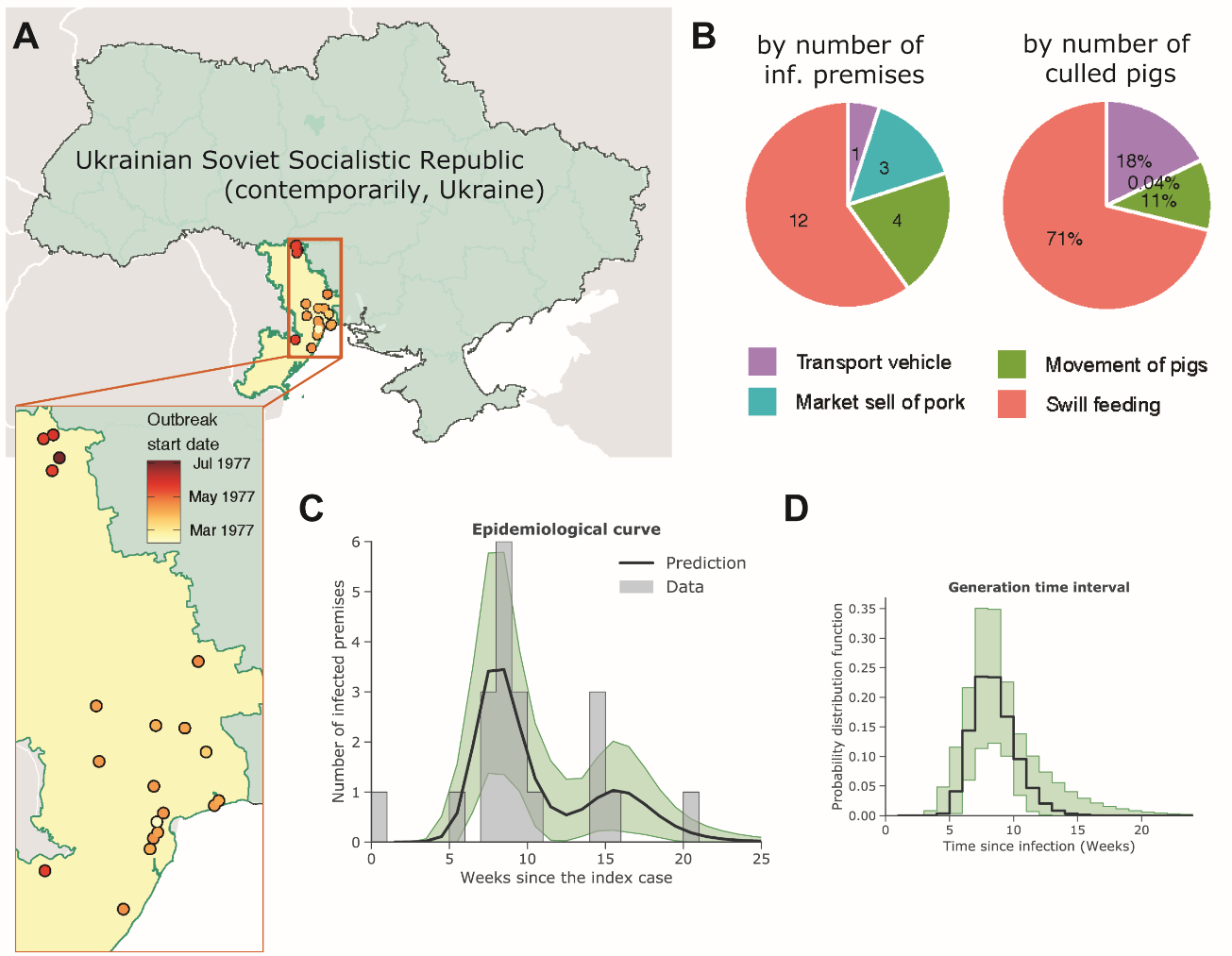
**

**Supplementary Figure 8: Trace plots of MCMC samples respective to the weekly number of cases (A) and generation time (B) show that both models with one and two generation of cases can be plausible.** The colour code indicates the varying values of $R_{2}$. When $R_{2}$ is close to zero, the lines are blue, and the lines are red for high values of $R_{2}.$ Solid lines correspond to the mean values.


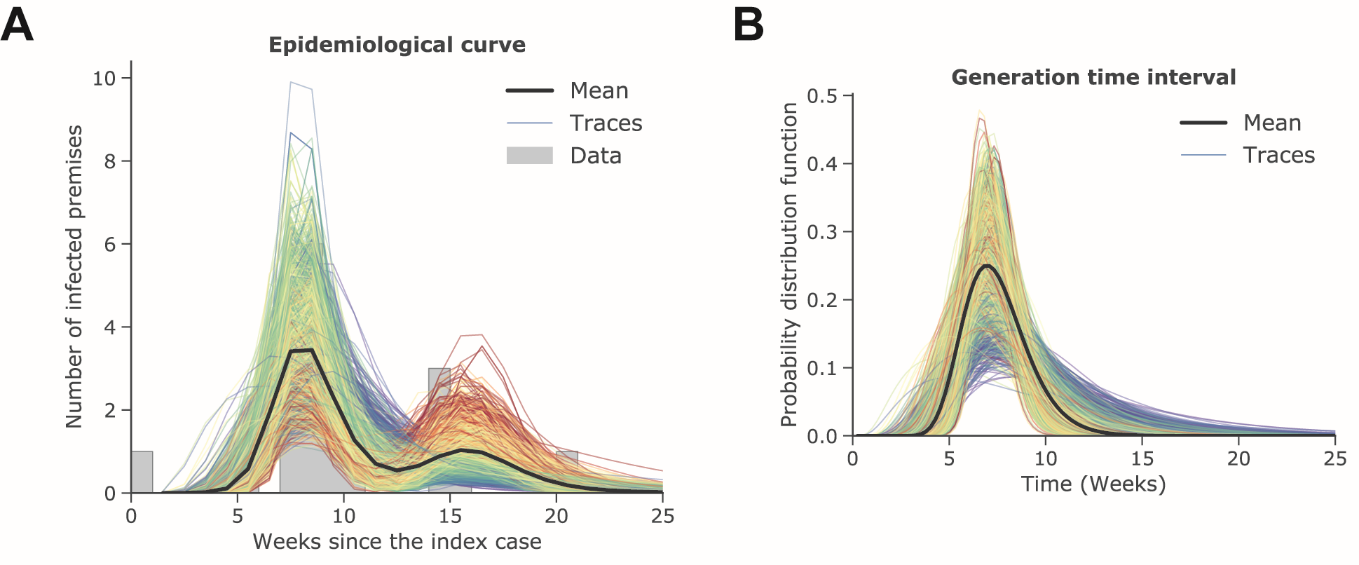


**Supplementary Materials**

1. **Inference of the generation time from historical records**

Estimation of the mean transmission distance using the method of Salje and colleagues [21] required knowledge of the generation time distribution of ASF between-farm transmission. As detailed investigation reports for the outbreaks in China were unavailable [48] we utilized an investigation report from an ASF outbreak in the Odessa region of the former Ukrainian Soviet Socialist Republic, the Union of Soviet Socialist Republics (USSR) in 1977 [29,30] to obtain a between-farm generation time distribution.

Prior to re-introduction of ASFV into post-USSR territory (Georgia) in 2007, the first and only epidemic of ASF in the USSR was in the Odessa region of the Ukrainian Soviet Socialist Republic, a part of contemporary Ukraine, in 1977 (**Supplementary Figure 7A**). The epidemic began around the end of February and continued until July. Twenty units were reported to be infected and a total of 360,500 pigs in epidemic zones and high-risk regions were culled, leading to a loss of 30.5% of pig herds in the region. The affected units ranged from small private backyards with only a few pigs to large collective farms with 1,509–13,865 pigs. The main driver of spread was insufficiently preheated swill feed (**Supplementary Figure 7B**).

Data were aggregated into weekly counts of infected units $\{n_{w};w=1,2,\ldots\}$ with the first week set to the date of start of the index outbreak. The force of infection $\lambda_{w}$ was modelled using a sequential generation process referred to as a generation-dependent model [31–33]. An infected unit generates new secondary infections based on the probability density function of the generation time $g_{w}(\boldsymbol{\theta}_{g})$. When a disease invades a new area, the index outbreak becomes the first generation. The index outbreak then generates $R_{1}$ new cases (second generation) with illness onset spread out over time according to the distribution $g_{w}$. The second generation cases $R_{1}$ subsequently generate $R_{2}$ new cases according to the same distribution $g_{w}$. We derived the expected total number of generated cases as: $R_{1}(g_{w}+R_{2}\left( g*g \right)_{w})$ using a maximum of three generations and assuming independent and identical transmission events. The symbol “$*$” stands for the convolution function, as described by:

$$\left( g*g \right)_{w}=\sum_{s=1}^{w-1} g_{w-s}\cdot g_{s}.$$

This method can be easily expanded to include a larger number of generations. We defined the force of infection in the following normalized form:

$$\lambda_{w}\left( \boldsymbol{\theta}_{\lambda}=\left\{ K,R_{2},\boldsymbol{\theta}_{g} \right\} \right)=K\frac{g_{w}+R_{2}\left( g*g \right)_{w}}{1+R_{2}},$$

where we eliminated $R_{1}$ from both the numerator and denominator as it was a common multiplier, and introduced a normalizing constant $K$ that represents the expected total number of infected units.

We identified the lognormal distribution as the best candidate distribution for $g_{w}$ among gamma, lognormal, and Weibull distributions by comparing Watanabe-Akaike Information Criterion values for each model fit with the lowest value preferred. We then used Bayesian inference for the generation-dependent model with a lognormally distributed generation time to estimate the model parameters $\boldsymbol{\theta}_{\lambda}$ including their uncertainty levels.

We determined that a three-generation process captured the dynamics of the epidemic sufficiently well (**Supplementary Figure 7C**), and the best-fitted lognormal distribution estimated the mean generation time at 7.54 weeks (95% CI: 6.60–8.90) with an SD of 1.73 weeks (95% CI: 0.92–3.06) (**Supplementary Figure 7D**). Our model fit also indicated that the between-farm reproduction number was below one for the second generation $R_{2}$, at which point the outbreak had been fully recognized by the authorities and control measures were implemented (median value: 0.43, 95% CI: 0–0.88).

**Supplementary Reference**

1. United States Department of Agriculture (USDA) Foreign Agricultural Service, 2019. GAIN Report: Chinese hog farmers take risks to restoke despite widespread African swine fever. 2019. [https://agriexchange.apeda.gov.in/marketreport/Reports/Livestock_and_Products_Annual_Beijing_China_Peoples_Republic_of_7-22-2019.pdf](https://agriexchange.apeda.gov.in/marketreport/Reports/Livestock_and_Products_Annual_Beijing_China_Peoples_Republic_of_7-22-2019.pdf%20Accessed%206%20August%202023) Accessed 6 August 2023
